# Biomarkers of coagulation, endothelial, platelet function, and fibrinolysis in patients with COVID-19: a prospective study

**DOI:** 10.1038/s41598-024-51908-9

**Published:** 2024-01-23

**Authors:** Manoj Job S.B., Binila Chacko, Sushil Selvarajan, John Victor Peter, Tulasi Geevar, Rutvi Gautam Dave, Josh Thomas Georgy, Anand Zachariah, Tina George, Sowmya Sathyendra, Samuel George Hansdak, Rajiv Karthik Krishnaswami, Balamugesh Thangakunam, Richa Gupta, Reka Karuppusami, Sukesh Chandran Nair, Alok Srivastava

**Affiliations:** 1 Department of Critical Care, Christian Medical College, Vellore, 632004 India; 2 Department of Clinical Hematology, Christian Medical College, Vellore, India; 3 Department of Transfusion Medicine, Christian Medical College, Vellore, India; 4 Department of General Medicine, Christian Medical College, Vellore, India; 5 Department of Infectious Diseases, Christian Medical College, Vellore, India; 6 Department of Pulmonary Medicine, Christian Medical College, Vellore, India; 7 Department of Respiratory Medicine, Christian Medical College, Vellore, India; 8 Department of Biostatistics, Christian Medical College, Vellore, India

**Keywords:** Prognostic markers, Viral infection, Pathogenesis

## Abstract

Prospective and sequential evaluation of homeostatic changes leading to thrombosis across COVID 19 disease severity spectrum are limited. In this prospective observational study, haemostasis was evaluated in patients with mild, moderate-severe, and critical COVID-19 infection. Markers of endothelial activation [Soluble thrombomodulin (sTM), von Willebrand Factor (VWF)], platelet activation [Soluble P-selectin, beta-thromboglobulin (BTG)] and global haemostasis [Rotational thromboelastometry (ROTEM)] were evaluated on days 1 and 5 after admission. The study cohort comprised of 100 adult patients (mild = 20, moderate-severe = 22, critical = 58). Sixty-five patients received anticoagulation for 10 (7–14) days. Thrombotic events were seen in 9 patients. In-hospital mortality was 21%. Endothelial activation markers were elevated at baseline in all subgroups, with levels in moderate-severe (sTM = 4.92 ng/ml, VWF = 295 U/dl) [reference-ranges: sTM = 2.26–4.55 ng/ml; Soluble P-selectin = 13.5–31.5 ng/ml; BTG = 0.034–1.99 ng/ml] and critical patients (sTM = 6.07 ng/ml, VWF = 294 U/dl) being significantly higher than in the mild group (sTM = 4.18 ng/ml, VWF = 206 U/dl). In contrast, platelet activation markers were elevated only in critically ill patients at baseline (Soluble P-selectin = 37.3 ng/ml, BTG = 2.51 ng/ml). The critical group had significantly lower fibrinolysis on days 1 and 5 when compared with the moderate-severe arm. COVID-19 infection was associated with graded endothelial activation and lower fibrinolysis that correlated with illness severity.

## Introduction

Since the onset of pandemic, SARS COV-2 infection has been associated with coagulation abnormalities and related increase in morbidity and mortality^1^. Increased incidence of DVT and PE were reported from patients with COVID 19 especially from patients admitted in ICU^2^. Furthermore, microvascular thrombosis was reported in pulmonary circulation contributing to the pathology of ARDS in COVID-19^3–5^.

The pathophysiological mechanism leading to these coagulation abnormalities has been theorised to result from dysregulated interaction between the coagulation system and immune system^6^. It is now known that coagulopathy in COVID 19 is not a simple disorder of one haemostatic component but a complex process affecting multiple pathways of haemostasis system such as coagulation, fibrinolytic, anticoagulation system which is in delicate balance with the endothelium to maintain haemostasis^7^. Studies prospectively evaluating these pathways using biomarkers across the disease severity spectrum are limited^8^.

**Figure 1:**
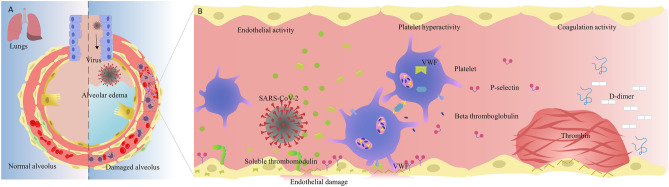
Pulmonary epithelial & endothelial damage, platelet activation and coagulopathy in COVID-19. (**A**) On the left, the normal alveolar and endothelial interface is depicted; the right side highlights the pathophysiology of severe SARS-CoV-2 infection in the lung which includes infection, inflammation, activation of endothelial, platelet and coagulation pathways, and alveolar oedema. (**B**) The virus infects and damages the pulmonary epithelial and endothelial cells, initiating a cascade of events such as endothelial activation—release of proinflammatory and prothrombotic factors (such as von Willebrand factor (VWF), thrombin and thrombomodulin), platelet hyperactivation—platelet micro vesicle and granule release of (P-selectin, beta thromboglobulin) into circulation and coagulation activation—increased clot formation leading to fibrinolysis and increased fibrin degradation products and D-Dimer.

This prospective study was undertaken to comprehensively evaluate the endothelial, platelet and global haemostasis changes (Fig. 1) at two time points during hospital admission to better understand their roles and assess if specific abnormalities correlated with clinical outcomes across the WHO severity spectrum^9^ of COVID-19 infection.

## Methods

### Study design and participants

This observational study was prospectively carried out in a large tertiary care university affiliated teaching hospital in South India. Hospitalized adult patients (> 18 years), with a polymerase chain reaction (PCR) confirmed diagnosis of COVID-19 were prospectively enrolled between October 2020 and November 2020. All study procedures were performed in accordance with the ethical standards for research with human participants and was approved by the Institutional Review Board and Ethics Committee of the hospital (IRB Min No 13392, dated 23.09.2020). Informed consent was obtained from the patient or their legal guardian.

**Figure 2:**
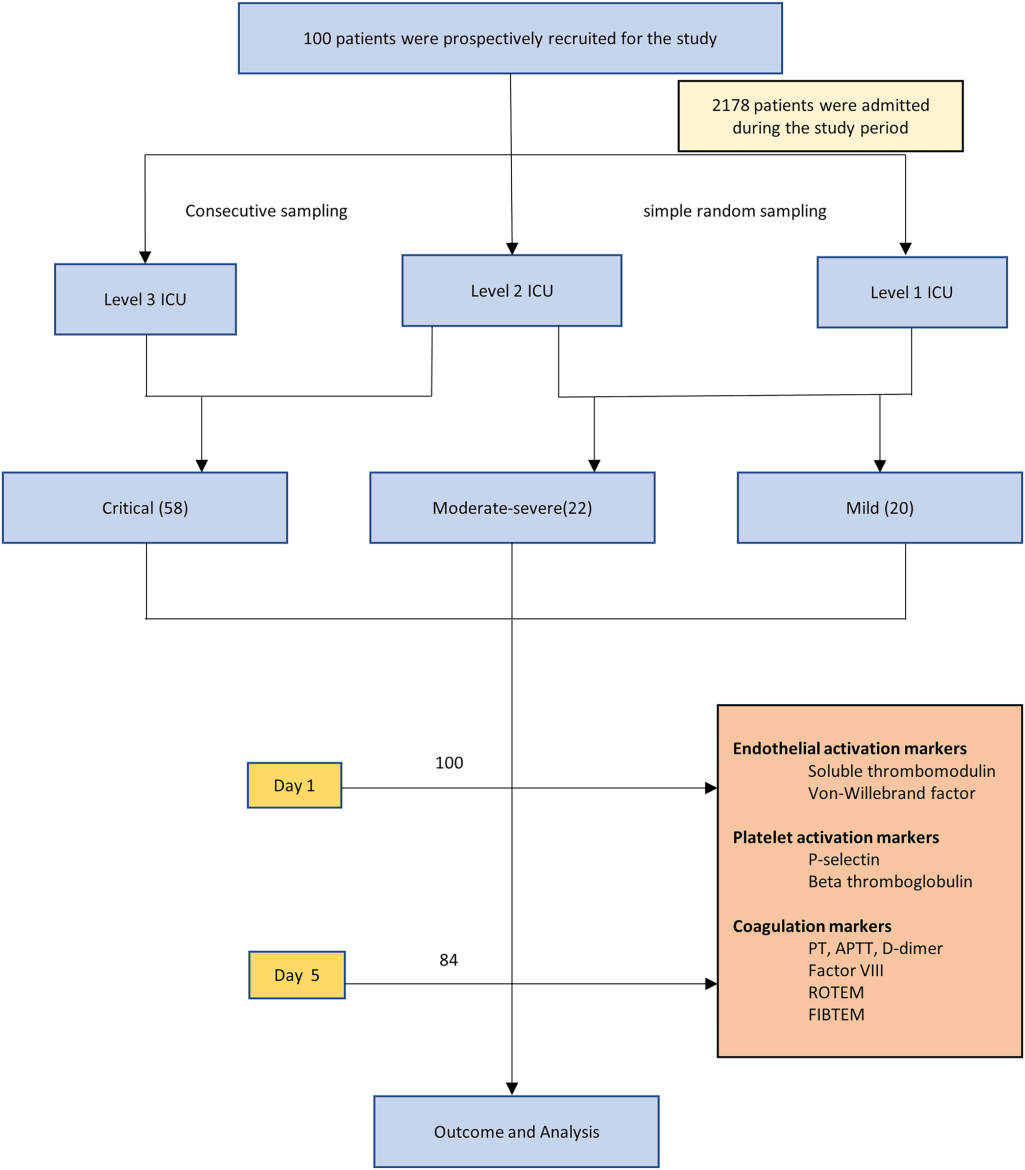
Strobe figure.

Patients were categorised at hospital admission as mild, moderate, severe and critical disease as per the World Health Organization (WHO) severity classification^9^. Based on disease severity and the hospital protocol, patients were admitted to different levels of care: *Level 1* for mild to moderate COVID-19 disease, *Level 2* for severe disease and *Level 3* (ICU) for the critically ill COVID-19 patients. As the pandemic progressed, due to shortage of ICU beds, there was a need to manage critical COVID-19 patients in the Level 2 wards as well. Consecutive critically ill patients admitted to the ICU and eligible non-critically ill patients in the wards were recruited (Fig. 2).

Demographic data, co-morbidities, treatment, and outcomes were recorded. Treatment included anti-viral therapy (remdesivir) and steroids (dexamethasone 6 mg once daily or equivalent doses of methyl prednisolone) for severe and critical category of COVID-19 patients with oxygen requirement. Steroids was started within 24 h when indicated. Anticoagulation (prophylactic or therapeutic) was initiated in all critically ill COVID-19 patients unless there were contraindications. Therapeutic anticoagulation was considered if D-dimer was > 1000 ng/ml in the setting of worsening respiratory status with or without proven thrombotic events. In the non-ICU setting, anticoagulation was administered as per clinician discretion, based on the available scientific evidence at that time. Chest CT scan was not routinely done for all patients and was performed only if the critical COVID-19 patients were safe for transfer.

### Laboratory evaluation

Blood for coagulation and ELISA based tests was collected in 3.2% trisodium citrate using the evacuated tube system; 2-ml ethylene diamine tetra acetic acid (EDTA) blood was collected for complete blood count. To minimise potential preanalytical activation of plasma biomarkers of platelet activation, all blood samples were collected by trained technical staff during the morning hours and were manually transported to the laboratory without the use of pneumatic tube system^10^. Upon arrival at the laboratory, the samples were centrifuged at 2500 g for 20 min to obtain platelet-poor plasma which was used for routine plasma-based tests, including PT, APTT, fibrinogen, factor VIII, and D-dimer, as well as whole-blood tests such as ROTEM and PFA-200, all completed within 4 h of sample collection. For Biomarker analysis and storage, the plasma samples were stored at − 80 °C in aliquots within 2–3 h of sample collection. Samples were thawed at a later date and the test completed in batches. Once thawed, samples were not reused. For all patients, measurement of coagulation, endothelial and platelet markers were done at recruitment and 5 days after recruitment.

Prothrombin time (PT), Activated partial thromboplastin time (APTT), fibrinogen and factor VIII were performed on platelet poor plasma on automated coagulation analyser, ACL Top 750 CTS (Instrumentation Laboratories (IL), Bedford, USA). Factor VIII was measured by the one-stage APTT based assay using factor VIII-deficient plasma (HemosIL, IL, Bedord, USA) and compared against calibrator (Unicalibrator, IL, Bedford, USA). D-dimer was measured by automated chemiluminescent immunoassay on ACL AcuStar (IL, Bedford, USA).

Endothelial activation markers that were evaluated included soluble thrombomodulin (sTM) and Von Willebrand Factor (VWF) antigen and markers of platelet activation comprised of Platelet Function Analyser (PFA 200, Siemens, Dade Behring, Germany), soluble P-selectin and beta-thromboglobulin (BTG). Soluble P-selectin, sTM and BTG were measured using ELISA assays (soluble P-selectin: Ray Biotech, USA, Lot No. 1202200217; soluble thrombomodulin: R&D Systems, USA, Lot No P253002; soluble beta-thromboglobulin: Fine Test, Wuhan, Batch No. H0874F121) according to manufacturer’s instructions on stored plasma. VWF antigen was measured using an in-house ELISA as described previously^11^. Testing was performed on Collagen/adenosine diphosphate (COL/ADP) cartridge on PFA-200 and closure time recorded. PFA was analysed as a marker of platelet hyperactivity based on the study by Yee et al.^12^ where it was found that healthy patients with a shortened CT on PFT had platelet hyperactivity based on additional tests, including an increase in aggregation (%) response in platelet aggregometry, surface P-selectin expression, and PAC-1 binding after agonist activation.

Viscoelastic testing was performed with citrated whole blood on Rotational Thromboelastometry, ROTEM (Tem International, Munich, Germany) using modified EXTEM and FIBTEM modes. EXTEM reagent with low tissue factor concentration was used to reflect physiological conditions^13^. The test was performed by trained personnel and activated using tissue factor (TF). The TF was prepared by dilution of PT reagent, Innovin (Dade Behring, USA) at 1:2000 dilution, modified from the method described by Sorenson et al.^11^ The variables assessed were clotting time (CT), clot formation time (CFT), alpha angle (α), maximum clot firmness (MCF), maximal lysis (ML). The reference ranges for these parameters were established using samples from over 300 blood donors. The influence of fibrinogen on clot firmness was estimated by using the platelet inactivating FIBTEM test as per manufacturer’s instructions. Complete blood count was done on EDTA blood on automated hematology analyser, DxH 900 (Beckman Coulter, Miami, Fl, USA).

### Sample size calculation and statistical analysis

Based on the study by Goshua et al.^14^, it was determined that we needed to recruit at least 47 SARS-CoV-2 patients each in the critically ill (critical COVID-19) and non-critically ill (mild, moderate and severe COVID-19) categories, assuming a mean difference of 1.2 ng/ml (SD_1_ = 2.89, SD_2_ = 0.44) in sTM levels between the 2 groups to achieve 80% power with a two-sided significance level of 0.05.

Continuous variables were presented as mean (standard deviation, SD) for normally distributed data and as median (interquartile range, IQR) for skewed data. Categorical data were reported as proportions and parametric t test or Mann Whitney U test were used for comparison as appropriate. For continuous variables three group comparison was performed using one way ANOVA or Kruskal Wallis test as appropriate. Student’s independent t-test was used for two group comparisons. Receiver operating characteristic (ROC) analyses were then performed using in-hospital mortality as the classification variable and biomarker levels at admission as the prognostic variable to determine the best cut off point for each of the markers. The optimal thresholds for each marker were determined by the highest Youden Index. Based on the best cut off, Kaplan–Meier curve were used to estimate the survival function from the time of admission to mortality and compared using log-rank tests. The Cox regression was performed to assess the factors associated with mortality and was reported as Hazard Ratio (HR) with 95% confidence interval (CI).

All tests were two-sided at α = 0.05 level of significance. All analyses were done using SPSS version 25.0 statistical package (IBM statistic, New York, USA) and SAS (version 9.1 for Windows; SAS Institute, Cary, NC, USA).

### Role of funding

Both internal funders (Institutional Internal Review Board and Department of Clinical Haematology) reviewed the study plan and streamlined study design, but had no role in data collection, laboratory testing, data entry and data analysis. No external funders sponsored this study.

## Results

### Baseline demographic data, treatment, and outcomes

One hundred patients with a mean (SD) age of 54.4 (14.1) years were prospectively recruited and categorized based on the WHO severity criteria as mild (n = 20), moderate-severe (n = 22) and critical (n = 58) COVID-19 infection (Table 1; Fig. 2). Critically ill patients were significantly older (*p* = 0.02) than those with mild disease, with a mean (SD) SOFA and APACHE-II scores of 3.98 (1.68) and 11.8 (4.87) respectively.11 patients were on aspirin prior to hospitalisation (Table 1). Lymphopenia at admission progressed as the disease severity increased. (supplementary Table S1).

**Table 1:** Patient Characteristics.

Parameter	Overall (n = 100)	Mild (n = 20)	Moderate-Severe (n = 22)	Critical (n = 58)	*p* value*	*p* value	*p* value
					All three group	Mild versus Moderate-Severe	Moderate-Severe versus Critical
Age, years^†^	54.4 (14.1)	46.8 (17.3)	55.7 (14.5)	56.6 (11.9)	**0.02**	0.08	0.81
Sex, male	78 (78%)	17 (85%)	17 (77.3%)	44 (75.9%)	0.69	0.70	0.89
Comorbidity^§^							
Comorbidities > 2	39 (39%)	5 (25%)	8 (36.4%)	26 (44.8%)	0.28	0.43	0.49
Malignancy	4 (4%)	2 (10%)	1 (4.5%)	1 (1.7%)	0.26	0.6	0.48
Thrombotic disorder	1 (1%)	0 (0%)	1 (4.5%)	0(0%)	0.18	1.00	0.28
Past drugs							
Aspirin	11 (11%)	2 (10%)	4 (18.2%)	5 (8.6%)	0.47	0.67	0.25
Anticoagulation	1 (1%)	0 (0%)	1 (4.5%)	0 (0%)	0.17	1.00	0.28
Onset of symptoms to hospital, days^‡^	4 (2–7)	2 (0–7)	5 (1–7)	4.5 (3–7)	0.10	0.22	0.59
Treatment data							
Remdesivir	55 (55%)	0 (0%)	4 (18.2%)	51 (87.9%)	** < 0.0001**	0.11	** < 0.0001**
Steroids	68 (68%)	1 (5%)	10 (45.5%)	57 (98.3%)	** < 0.0001**	**0.004**	** < 0.0001**
Use of anticoagulants	65 (65%)	1 (5%)	9 (40.9%)	55 (94.8%)	** < 0.0001**	0.01	** < 0.0001**
Therapeutic	57 (57%)	0 (0%)	3 (13.6%)	54 (93.1%)	N/A	N/A	N/A
Prophylactic	8 (8%)	1 (5%)	6 (27.3%)	1 (1.7%)	N/A	N/A	N/A
Duration of anticoagulation, days^‡^	10 (7–14)	5 (5–5)	6.5 (4–7.74)	11 (7.7–15.0)	N/A	N/A	0.01
Duration of ventilation^‡^	7.5 (6–14)	0	0	7.5 (6–14)	N/A	N/A	N/A
Ventilation free days, days^†^	17.4 (6.8)	0	0	17.4 (6.8)	N/A	N/A	N/A
Complications							
Thrombotic complications	9 (9%)	0 (0%)	6 (27.3%)	3 (5.2%)	N/A	N/A	N/A
Bleeding complications	0(0%)	0 (0%)	0 (0%)	0 (0%)	N/A	N/A	N/A
Acute kidney injury	19(19%)	1 (5%)	2 (9.1%)	16 (27.6%)	0.04	1.00	0.13
Blood stream infections^§§^	8 (8%)	0 (0%)	1 (4.5%)	7 (12.1%)	0.18	1.00	0.43
Ventilator associated infections	14 (14%)	0 (0%)	0 (0%)	14 (24.1%)	0.01	N/A	0.03
Outcome							
Hospital mortality	21 (21%)	0 (0%)	2 (9.1%)	19 (32.8%)	**0.002**	0.49	0.04
Hospital length of stay, days^‡^	10 (7–17)	7 (5–8.75)	7 (5–9)	14.5(10–22)	** < 0.0001**	0.23	< 0.0001

Data are presented as number (percentage) and *p* value is obtained from Chi-square test.

*p value is obtained from Chi-square test/Fisher’s exact test (less cell count) and Yates continuity correction (zero cell) for categorical data, one-way ANOVA for continuous data and Kruskal Wallis test for skewed data.

^†^ Data are presented as mean (SD) and *p* value is obtained from t test.

^‡^Data are presented as median (IQR), and *p* value is obtained from nonparametric Mann–Whitney U test.

^**§**^ Comorbidities include diabetes mellitus, hypertension, chronic heart disease, chronic kidney disease, chronic pulmonary disease.

^§§^Blood stream infections refer to cases of clinical sepsis confirmed by the presence of microorganisms in blood or sputum culture.

N/A: *p* value is not applicable due to very less number in one category/data is not available for one or two or three groups.

Significant values are in bold.

Overall, 65 patients were anti-coagulated; 57 [critical (n = 54), moderate-severe (n = 3)] received therapeutic anticoagulation with enoxaparin at 1 mg/kg twice daily and 8 patients [moderate-severe (n = 6), critical (n = 1) and mild (n = 1)] received intermediate-dose anticoagulation with enoxaparin at 1 mg/kg once daily for a median (IQR) duration of 11 (7.7–15) days. None of the patients had major or minor bleeding complications.

Thrombotic complications were seen in 9 patients. Five patients had evidence of thrombosis at presentation to the hospital [Cerebrovascular accident (n = 3), pulmonary embolism (n = 1), coronary event (n = 1)]. The remaining 4 patients, 3 of whom were on anticoagulation therapy, developed thrombotic complications during their hospitalisation [deep vein thrombosis (n = 2) and coronary event (n = 2)].

All patients in the critical category required ventilation [non-invasive ventilation only (n = 39) or invasive (n = 19)] for a median (IQR) duration of 7.5 (6–14) days. The critical COVID-19 subgroup had significantly longer median (IQR) duration of hospital stay as compared to the other categories (Table 1). The median (IQR) length of stay for the cohort was 10^7–17^ days. Hospital mortality was 21% (19 in the critical group, 2 in the moderate-severe group).

### Laboratory data on days 1 and 5

#### Endothelial activation markers

Baseline (day1) sTM was elevated above the normal reference range in the moderate-severe and critical groups, whereas VWF was elevated in all three severity groups and between group difference was significant (sTM *p* < 0.05 and VWF *p* < 0.0001). On further analysis, both VWF (*p* < 0.0001) and sTM (*p* = 0.003) had significantly higher values in the critical group as compared to the patients with mild disease. There was however no significant difference in VWF (*p* = 0.97) and sTM levels (*p* = 0.33) between the moderate-severe and the critical groups (Table 2**).**

**Table 2:** Endothelial, platelet and coagulation markers at day 1 and 5.

Parameter	Day 1							
	Reference ranges	Mild (n = 20)	Moderate-severe (n = 22)	Critical (n = 58)	*p* value*	*p* value		
					All three group	Mild versus Moderate-Severe	Moderate-Severe versus Critical	Critical versus Mild
Endothelial activation markers								
Soluble thrombomodulin, ng/mL	2.26–4.55	4.18 (1.62)	4.92 (1.78)	6.07 (3.87)	**0.05**	0.17	0.33	**0.003**
VWF Ag, U/dL	61.3–157.8	206 (79.5)	295 (98.7)	294 (79.2)	** < 0.0001**	**0.003**	0.97	** < 0.0001**
Platelet number and activation markers								
Platelet Count, 10^9^ cells per L	150–400	203.8 (88.5)	197 (99.2)	163.3 (76.9)	0.10	0.82	0.11	0.054
PFA 200, s	68–142	106 (53.6)	104 (43.9)	114 (58.8)	0.92	0.90	0.46	0.590
Soluble P-selectin, ng/mL	13.5–31.5	21 (8.59)	23.9 (18.7)	37.3 (19.5)	** < 0.0001**	0.53	** < 0.0001**	**0.001**
Beta-thromboglobulin, ng/mL	0.034–1.99	0.81 (0.48)	0.74 (0.55)	2.51 (3.34)	** < 0.0001**	0.31	** < 0.0001**	**0.027**
Coagulation parameters								
Prothrombin time, s	8–11.2	10.6 (1.53)	10.8 (2.07)	12.2 (3.80)	0.07	0.70	0.11	0.071
APTT, s	25.2–38	33.7 (5.32)	32.6 (6.00)	35.4 (11.6)	0.56	0.51	0.34	0.619
D-dimer, ng/mL^†^	< 250	325(225–724)	653(375–1718)	1155(547–3425)	** < 0.0001**	**0.04**	0.09	** < 0.0001**
Fibrinogen, mg/dL	150–300	365 (109)	346 (132)	389 (149)	0.44	0.62	0.24	0.509
Factor VIII, U/dL	50–150	96.6 (33.6)	114 (35.8)	118 (37.7)	0.09	0.12	0.66	**0.03**
Parameter	Day 5							
Endothelial activation markers								
Soluble thrombomodulin, ng/mL	2.26–4.55	3.45 (1.73)	4.59 (1.46)	6.66 (3.96)	** < 0.0001**	**0.01**	0.07	** < 0.0001**
VWF Ag, U/dL	61.3–157.8	191 (89.7)	265 (88.0)	317 (88.0)	** < 0.0001**	**0.03**	**0.05**	** < 0.0001**
Platelet number and activation markers								
Platelet Count, 10^9^ cells per L	150–400	240 (95.2)	244 (11.5)	188 (78.1)	**0.05**	0.92	**0.03**	**0.034**
PFA 200, s	68–142	118 (56.7)	111 (44.9)	88.5 (41.5)	**0.04**	0.67	0.07	**0.026**
Soluble P-selectin, ng/mL	13.5–31.5	25.7 (11.5)	25.3 (14.6)	45.7 (19.9)	** < 0.0001**	0.80	** < 0.0001**	** < 0.0001**
Beta-thromboglobulin, ng/mL	0.034–1.99	0.82 (0.57)	0.80 (0.63)	2.56 (3.30)	** < 0.0001**	0.84	** < 0.0001**	**0.047**
Coagulation parameters								
Prothrombin time, s	8–11.2	10.3 (1.32)	10.4 (2.03)	13.0 (4.86)	**0.02**	0.91	**0.04**	**0.038**
APTT, s	25.2–38	32.5 (4.05)	33.6 (7.05)	38.9 (16.1)	0.16	0.89	0.21	0.136
D-dimer, ng/mL^†^	< 250	39 (265–1516)	780 (470–1208)	1304 (710–3196)	**0.004**	0.24	**0.02**	**0.005**
Fibrinogen, mg/dL	150–300	446 (118)	388 (132)	396 (131)	0.37	0.21	0.84	0.187
Factor VIII, U/dL	50–150	141 (85.2)	162 (98.8)	135 (38.9)	0.34	0.54	0.11	0.791

Data are presented as mean (SD) and *p* value is obtained from t test.

^†^Data are presented as median (IQR) and *p* value is obtained from non-parametric Mann–Whitney U test. VWF Ag, von Willebrand factor antigen; PFA 200, platelet function analysis; APTT, Activated partial thromboplastin time; CT, Clotting time; CFT, Clot formation time; MCF, Maximum clot firmness; ML, maximal lysis. †† EXTEM reagent was modified by using low tissue factor to concentration to reflect physiological conditions.

*p value is obtained from one-way ANOVA for continuous data and Kruskal Wallis test for skewed data.

Significant values are in bold.

By Day 5, both sTM and VWF levels decreased or remained the same in the mild group when compared to baseline. Moderate-severe and critical patients continued to have elevated levels and there was significant between group difference across all 3 categories (sTM and VWF *p* < 0.0001) (Table 2).

#### Platelet activation markers

P-selectin and beta-thromboglobulin were elevated only in the critically ill patients on days 1 and 5 (Table 2) and these were significantly higher when compared with the mild and moderate-severe group of patients.

#### Coagulation parameters

Baseline D-dimer was elevated in all three severity groups above the reference range (Table 2). On day 1, moderate-severe (*p* = 0.04) and critical patients (*p* < 0.0001) had significantly higher D-dimer values in comparison to the mild group. By day 5, D-dimer continued to remain elevated over the reference range; the critical group had significantly increased D-dimer values over the moderate-severe (*p* = 0.02) and mild (*p* = 0.005) groups of patients.

Increased fibrinogen levels were seen in all 3 groups on days 1 and 5 above the reference range, albeit not statistically different among the 3 groups. In this cohort, there were no differences noted in factor VIII levels between the groups (Table 2).

#### Global haemostasis

Among ROTEM parameters, shorter clot time was observed in the mild group as compared to the moderate-severe and critical arms on both day 1 (*p* = 0.007) and day 5 (*p* = 0.01). The critical group had a lower percentage of clot lysis on days 1 and 5 (*p* = 0.003 and < 0.0001 respectively) when compared with the moderate-severe arm (supplementary Table S2).

### Laboratory markers and hospital mortality

**Figure 3:**
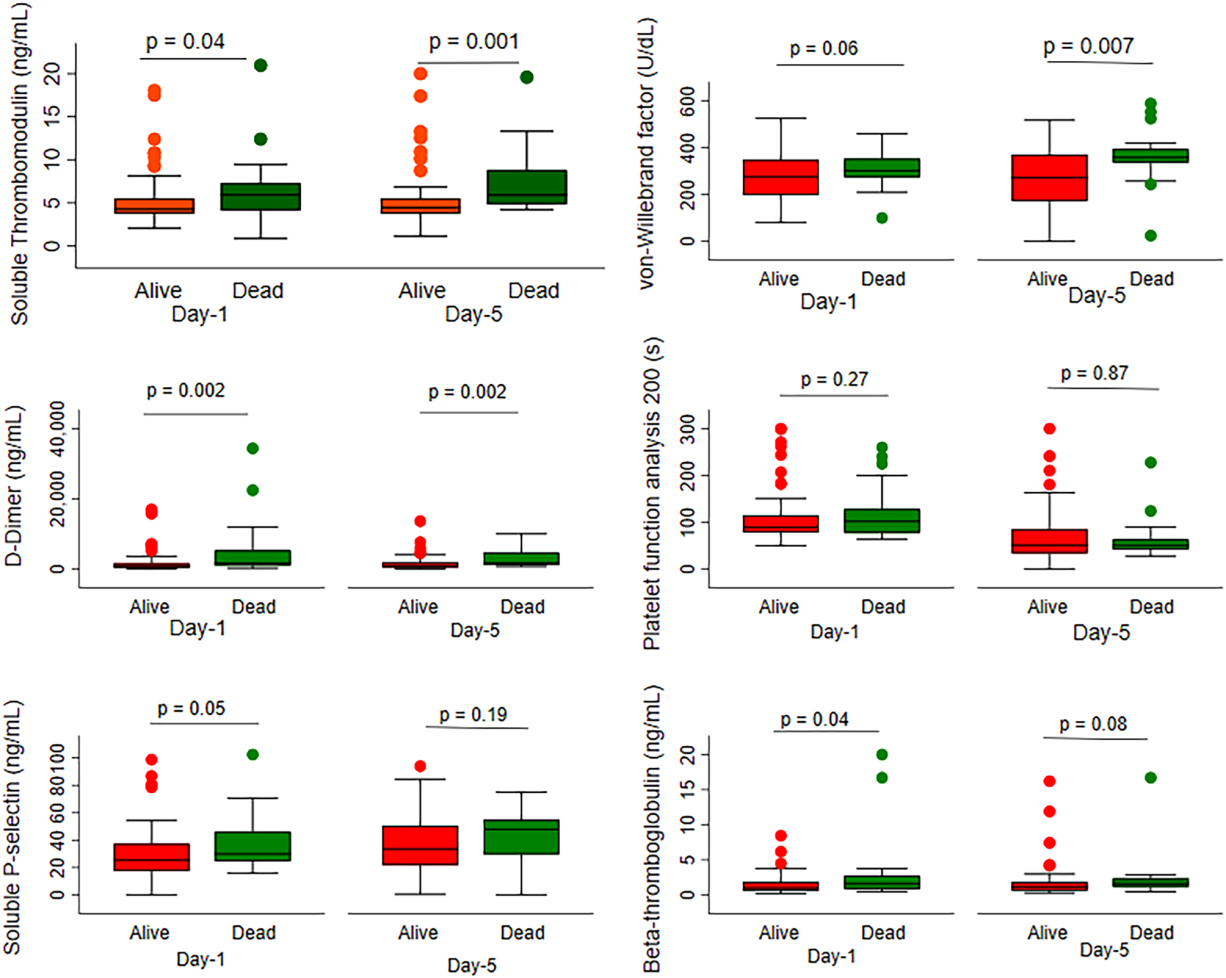
Comparisions of endothelial, platelet and coagulation markers using Box-whisker plots among the survivors and non-survivors at day 1 and day 5.

In the entire cohort, Day 1 sTM (*p* = 0.04), D-dimer (*p* = 0.002), soluble P-selectin (*p* = 0.05), BTG (*p* = 0.04), and Day 5 sTM (*p* = 0.001), D-dimer (*p* = 0.002) were significantly higher in the deceased patients (Fig. 3).

Differences between deceased with ICU and non-ICU survivors were further explored. When compared with the non-ICU survivors (patients admitted in the wards), the deceased patients had significantly elevated baseline (day1) sTM (*p* = 0.01), VWF (*p* = 0.007), Soluble P-selectin (*p* = 0.001), BTG (*p* < 0.001), D-dimer (*p* < 0.001) levels and day 5 sTM (*p* < 0.001), VWF (*p* < 0.001), Soluble P-selectin (*p* = 0.005), BTG (*p* < 0.001) and D-dimer (*p* < 0.001) and lower fibrinolysis (*p* = 0.001). Increased D-dimer and Factor VIII levels on day 5 [*p* = 0.05 and *p* = 0.03] were associated with increased mortality in the critically ill group. There was however no significant difference in the endothelial and platelet activation markers between ICU survivors and the deceased (Table 3).

**Table 3:** Comparison of Laboratory markers with mortality at two time points.

	Reference ranges	Day 1					Day 5				
		Non-ICU alive (n = 40)	ICU alive (n = 39)	Dead (n = 21)	*p* value		Non-ICU alive (n = 38)	ICU alive (n = 30)	Dead (n = 16)	*p* value	
					Non-ICU alive versus dead	ICU alive versus dead				Non-ICU alive versus dead	ICU alive versus dead
Endothelial activation markers											
Soluble thrombomodulin, ng/mL^‡^	2.26–4.55	4.0 (3.5–5.0)	4.5 (3.8–6.2)	5.9 (3.9–7.5)	**0.01**	0.19	3.7 (3.3–4.6)	4.8 (4.2–6.0)	5.9 (4.8–8.9)	** < 0.001**	0.07
VWF antigen, U/dL	61.3–157.8	245 (96.4)	291 (81.7)	309 (78.1)	**0.007**	0.39	224 (93.8)	307 (80.3)	345 (99.6)	** < 0.001**	0.15
Platelet number and activation markers											
Platelet Count, 10^9^ cells per L	150–400	203 (94.7)	171 (76.1)	147 (74.4)	**0.02**	0.24	242 (106)	201 (77.4)	159 (71.9)	**0.003**	0.07
PFA 200, s	68–142	106 (49.1)	109 (57.1)	122 (60.0)	0.29	0.4	114(51)	85.9 (40.8)	96.3 (42.8)	0.21	0.41
Soluble P-selectin, ng/mL^‡^	13.5–31.5	19 (15.8–27.4)	32.7 (22–47.3)	30 (22.4, 49.1)	**0.001**	0.83	23.7 (17.4–30.1)	42.8 (34.1–56.6)	47.9 (27.3–55.6)	**0.005**	0.89
Beta-thromboglobulin, ng/mL^‡^	0.034–1.99	0.6 (0.5–0.9)	1.8 (1.0–2.4)	1.6 (0.8–2.8)	** < 0.001**	0.76	0.6 (0.4–0.9)	1.7 (1.1–2.4)	1.6(1.1–2.4)	** < 0.001**	0.96
Coagulation parameters											
Prothrombin time, s	8–11.2	10.7 (1.85)	12.2 (4.39)	11.9 (2.2)	**0.04**	0.73	10.3 (1.7)	12.1 (1.9)	15 (8.1)	**0.03**	**0.04**
APTT, s	25.2–38	33.3 (5.7)	35.1 (13.7)	34.7 (5.4)	0.35	0.89	32.9(5.8)	35.6 (6.6)	46.5 (26.4)	0.06	**0.02**
D-dimer, ng/mL^†^	< 250	433 (288–804)	817 (477–2831)	1684 (840–5960)	** < 0.001**	0.07	545 (335–1073)	1100 (557–2913)	1809 (1113–5173)	** < 0.001**	**0.05**
Fibrinogen, mg/dL	150–300	358 (123)	381 (152)	393 (143)	0.35	0.79	420 (126)	408 (131)	359 (129)	0.13	0.21
Factor VIII, U/dL	50–150	105 (36.1)	122 (40.5)	110 (29.5)	0.50	0.27	153 (92.9)	142 (33.3)	117 (45.5)	0.09	**0.03**
Global hemostasis											
MODIFIED EXTEM^††^											
CT, s^†^	324–565	310 (259–381)	375 (321–501)	354 (279–397	0.29	0.22	311 (263–347)	362 (300–460)	483 (315–698)	**0.002**	0.07
CFT, s^†^	112–224	96 (79.5–131)	96 (80–182)	103 (90–128)	0.39	0.88	93 (76–104)	106(85–146)	133 (92–283)	**0.01**	0.17
Alpha angle, degree	50–68	69.2 (8.1)	65.1 (13.3)	67.9 (8.4)	0.55	0.39	71 (7.6)	66.1 (12.8)	58.2 (19.1)	**0.02**	0.08
MCF, mm	55–66	65.1 (8.7)	65.2 (9.4)	64 (8.3)	0.63	0.62	67.2 (7.8)	65.1 (9.1)	61.4 (11.5)	0.09	0.23
ML, %^†^	0–15	6.5 (4–11)	4 (2–7)	5 (2–10)	0.08	0.79	8 (5–10)	3 (1–5.2)	1 (0–5.7)	**0.001**	0.24
FIBTEM											
CT, ^†^	233–426	331 (266–372)	383 (329–507)	346 (288–393)	0.29	0.10	322 (280–364)	381 (333–468)	528 (367–699)	**0.001**	0.09
MCF, mm	4.4–18.8	27.8 (13.6)	24.8 (9.1)	25.9 (8)	0.49	0.67	29.1 (14.2)	22.9 (8.1)	22.5 (7)	**0.04**	0.88

Data are presented as mean (SD) and *p* value is obtained from t test.

VWF Ag, von Willebrand factor antigen; PFA 200, Platelet function analysis; APTT, Activated partial thromboplastin time; CT, clotting time; CFT, Clot formation time; MCF, maximum clot firmness; ML, Maximal lysis. ††EXTEM reagent was modified by using low tissue factor concentration to reflect physiological conditions.

^†^Data are presented as median (IQR), and *p* value is obtained from non-parametric Mann–Whitney U test.

Significant values are in bold.

**Figure 4:**
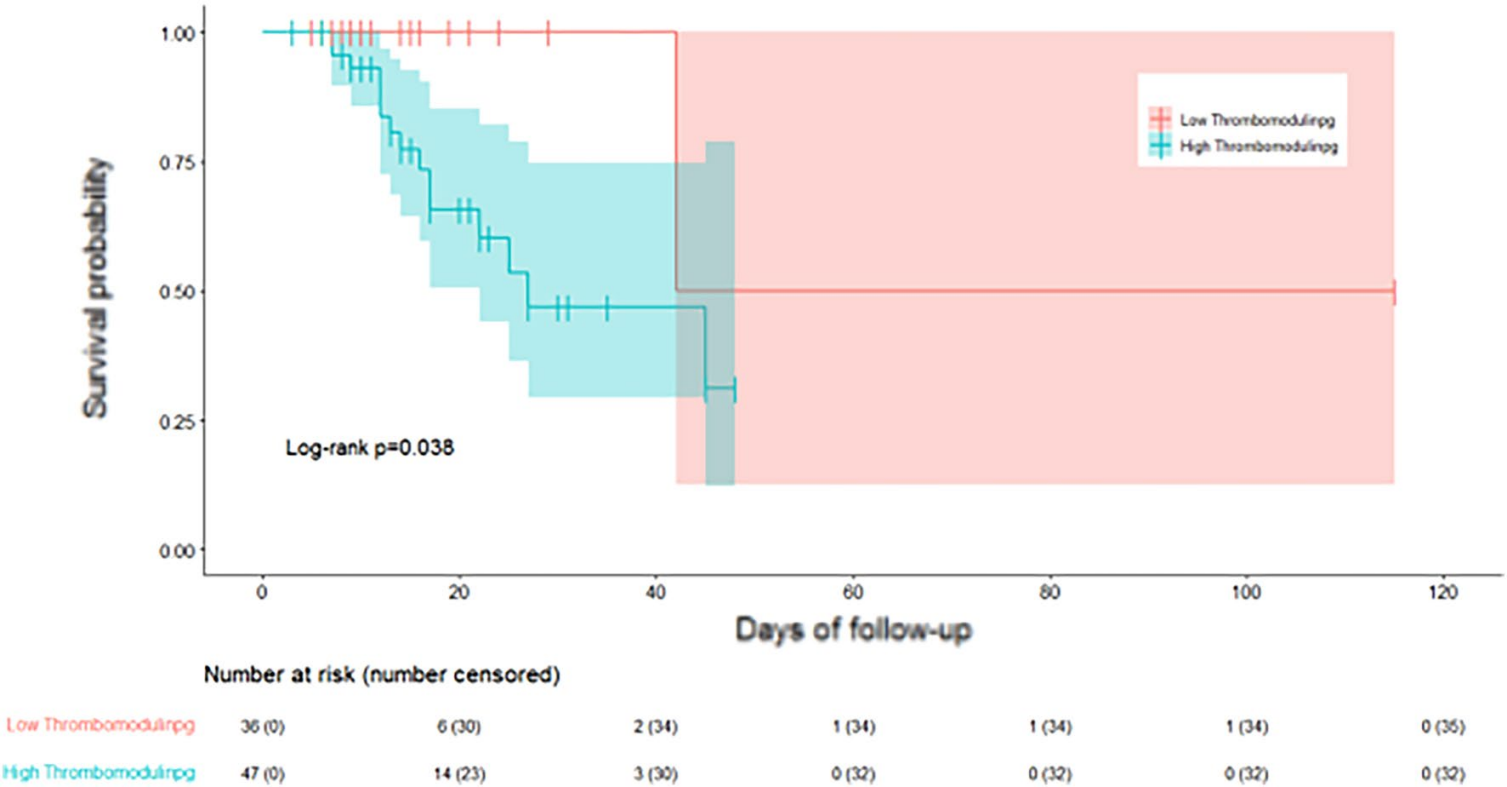
Kaplan Meier curve of survival and soluble throbomodulin levels on day 5.

Cox regression analysis showed that baseline (day 1) markers did not predict mortality; Day 5 sTM > 4.6 ng/ml [hazard ratio (HR) 6.4, 95% CI 0.84–48.1), *p* = 0.07] and day 5 D-dimer > 1103 ng/ml [HR 3.84, 95% CI (0.86, 17.08), *p* = 0.078] showed a trend towards increased hospital mortality (supplementary Table S3). In the survival analysis, hospital mortality was significantly lower among patients with day 5 sTM levels < 4.6 ng/ml (*p* = 0.04) (Fig. 4).

## Discussion

The mechanisms driving thrombosis in COVID-19 comprise a complex relationship between SARS CoV-2 infection and local/systemic haemostatic balance, which is not fully explained by the traditional risk factors for thrombosis. This prospective study systematically analysed endothelial, platelet and coagulation activation across the entire spectrum of COVID-19 disease severity and in-hospital mortality. Thrombotic events were seen in 9 patients and in-hospital mortality was 21%. There was graded increase in endothelial activation markers with increasing severity of COVID-19. Higher d-dimer values and lower fibrinolysis were also associated with increasing severity and mortality. In contrast, increased platelet activation, even though an early marker of thrombo-inflammatory response, was evident only in the critically ill, both at baseline and 5 days after recruitment. Unless there was a contraindication, all critically ill patients were on therapeutic anticoagulation. This could have resulted in increased clot times in the critical group. Additionally, lower clot lysis was found in the critical COVID-19 group.

Limited evidence suggested that endotheliopathy is a prominent feature in the pathogenesis of hypercoagulability in severe COVID-19 infection^14–17^. Our data shows that endothelial activation occurs across the spectrum of SARS-CoV2 severity at both time points. Whilst baseline VWF was significantly elevated in the three COVID-19 subgroups, sTM was significantly elevated in the moderate-severe and critical group. By day 5, these values decreased in the mild and moderate-severe groups but remained persistently elevated and on the increasing trend in the critical group. It is uncertain why, unlike similar studies^14,17^, the critical group of patients in our study did not have markedly increased VWF levels. It is possible that the early initiation of therapeutic anticoagulation in 95% of the critically ill patients may have contributed to comparatively lower VWF levels^18^. In addition to the previously reported observations of abnormal endothelial activation characterised by elevated sTM and VWF levels in the critically ill^14^, this study has demonstrated that VWF was elevated even in the mild COVID-19 patient group-suggesting that endotheliopathy may be an early feature in this disease. Given the recent reports of the role of persistent endotheliopathy in the pathogenesis of late COVID-19 related complications, it will be important to follow-up on these patients with early dysfunction and evaluate for the altered VWF-ADAMTS-13 axis as well as the reported abnormalities in monocytes and T cells^19,20^. While it can be argued that VWF is not a pure endothelial marker since it can be released by platelets and megakaryocytes, there is research^21,22^ to indicate that VWF can be used as a marker for endothelial injury and thrombotic risk in COVID 19.

In contrast to the endothelial markers, platelet activation markers, characterised by P-selectin and BTG, were elevated only in the critically ill. To the best of our knowledge, this is the first study that assessed beta thromboglobulin levels in SARS-CoV2 infection. This marker has been previously studied in settings of cerebrovascular accidents and ischemic heart disease^23,24^. Subsequent measurement of these markers on day five revealed a similar pattern. While Comer et al.^25^ found increased P-selectin both in severe and non-severe forms of COVID-19, this study found significantly higher P-selectin levels only in the critical group. It is unclear why these markers were not elevated in the mild and moderate disease categories since one would expect platelet activation markers to parallel the endothelial and coagulation hyperactivity^25,26^. As alluded to by Comer et al.^25^ this study raises the possibility of an alternate trigger for platelet activation.

Documenting laboratory evidence of hypercoagulability and impaired fibrinolysis has been a challenge in critical care. While viscoelastometry tests (VET) provide a global assessment of dynamic process of blood clot formation and lysis, evidence for its clinical usefulness in stratifying risk for thrombosis and adjustment of thromboprophylaxis in the critically ill is limited. Similar to other VET studies on COVID-19^27–29^ this study detected the presence of hypercoagulability even in mild COVID-19 disease, characterised by shortened clot time (CT), clot formation time (CFT), increased alpha angle and maximum clot firmness (MCF). FIBTEM MCF was also elevated suggesting increased contribution from fibrinogen towards clot formation. Despite the critically ill subgroup being on therapeutic anticoagulation, CT and CFT were within the normal range, suggesting a possible hypercoagulable state. In line with several studies that have reported hypo-fibrinolysis in COVID-19^30^, this study found significantly lower maximum lysis in the critical group. Normally, endothelial fibrin accumulation is prevented from progressing to microvascular thrombosis by fibrinolytics such as tissue plasminogen activator (tPA) and urokinase. Mechanisms driving lower fibrinolysis or fibrinolysis shutdown in critically ill could be due to overwhelming levels of plasminogen activator inhibitor 1(PAI-1) and thrombin activable fibrinolysis inhibitor (TAFI) resulting in lower fibrinolysis^31^.

Even though the non-survivors had higher endothelial and platelet markers when compared with the patients who did not require ICU, these markers did not help predict mortality in the critically ill. One reason for this is that our study was not powered to answer this question. A larger study may be required to clarify this issue. Relevantly, previous studies report sTM levels^14^ and VWF^32^ levels correlated significantly with mortality. Additionally, differences in spectrums of severity of disease among our patients included and the use of therapeutic anticoagulation in most of the patients in this study may also have led to the lack in predictive significance for mortality of these markers. The observed trend of increased mortality in conjunction with elevated levels of thrombomodulin and d-Dimer on day 5 may be attributable to disease progression, occurrence of micro-thrombosis in intervening period, increasing systemic inflammation due to COVID-19 or secondary infections. Additional research is required to comprehend this association.

Prior research has not analysed multiple haemostatic pathways but instead focused on individual markers^8,14,21,30,32^. Additionally, most of these research has been done using retrospective or cross-sectional methods^21,30,32^. While most studies have recorded these parameters at baseline, Pavoni et al.^28^ and Correa et al.^33^ analysed serial samples which included only critical COVID patients. This is study is unique in that it has prospectively and simultaneously analysed the various haemostatic pathways at two time points across the entire WHO severity spectrum in COVID-19 infection. This study has demonstrated that there is abnormal endothelial activation even in the milder forms of disease, whereas platelet activation markers were elevated only in the critical group suggesting a potential role of prophylactic antiplatelet therapy in the moderate-severe category.

Limitations of this study include the relatively small sample size, potentially significant clinical heterogeneity of patients within each severity strata, inherent systemic biases, and data from a single centre study. Given the pragmatic nature of the study conducted in the peak of COVID-19 baseline samples were collected after the first dose of anticoagulation. However, all samples were collected within 24 h of first dose of anticoagulation and variations within this window could be a confounder for our analysis. In addition, the use of therapeutic anticoagulation especially in the patients with critical illness may have mitigated haemostatic markers of COVID-19 associated coagulopathy and endotheliopathy. Also, more detailed testing and studies will be needed to further ascertain the potential effects of circulating thrombomodulin on activated protein C and thrombin-activable fibrinolysis inhibitor as these were not investigated in this study. Global coagulation testing was restricted to visco-elastic testing and further tests including thrombin generation assay and clot waveform analyses could be done to further assess in this regard. Not to mention, as this study was done during the first wave there may be differences with subsequent waves due to other variants^34^.

## Conclusion

In conclusion, the analysis of the haemostatic components across the spectrum of severity of COVID-19 disease demonstrated dysregulated endothelial function with increasing illness severity. Hypercoagulability and impaired fibrinolysis were also noted. Increased platelet activation was evident only in the critically ill, suggesting its importance both as biomarker and as a possible target in the management of more severe disease presentations. Although there was a significant difference in these markers among non-ICU survivors and those who succumbed to critical illness, larger studies will be required to ascertain if these biomarkers may predict survival.

### Supplementary Information

Supplementary Tables.

## Data Availability

The datasets used and/or analysed during the current study available from the corresponding author on reasonable request.

## Abbreviations

APTT: Activated partial thromboplastin time
BTG: Beta-thromboglobulin
CFT: Clot formation time
COL/ADP: Collagen/adenosine diphosphosphate
CT: Clotting time
ICU: Intensive care unit
MCF: Maximum clot firmness
ML: Maximal lysis
PAI: Plasminogen activation inhibitor
PCR: Polymerase chain reaction
PFA: Platelet function analyser
PT: Prothrombin time
ROTEM: Rotational thromboelastometry
sTM: Soluble thrombomodulin
TAFI: Thrombin activable fibrinolysis inhibitor
TF: Tissue factor
VET: Viscoelastometry tests
VWF: Von Willebrand factor
WHO: World Health Organization
