# Supplementary material for: Biomarkers of coagulation, endothelial, platelet function, and fibrinolysis in patients with COVID-19: a prospective study
Source: Sci Rep. 2024 Jan 23;14:2011. doi: 10.1038/s41598-024-51908-9 (PMC10805716; doi:10.1038/s41598-024-51908-9)
Supplement: Supplementary file 1 — Supplementary Tables. [file 41598_2024_51908_MOESM1_ESM.docx]

**Supplementary Table S1:** Additional Patient Characteristics

|  | **Overall (n=100)** | **Mild**  **(n=20)** | **Moderate-Severe (n=22)** | **Critical (n=58)** | **p value^*^** | **p value** | **p value** |
| --- | --- | --- | --- | --- | --- | --- | --- |
| Parameter |  |  |  |  | **All three group** | **Mild vs Moderate-Severe** | **Moderate-Severe vs Critical** |
| **Signs, symptoms and investigations** | | | | | | | |
| Fever | 73 (73%) | 14 (70%) | 14 (63.6%) | 45 (77.6%) | 0.43 | 0.66 | 0.21 |
| Breathlessness | 56 (56%) | 2 (10%) | 7 (31.8%) | 47 (81%) | **<0.0001** | 0.14 | **<0.0001** |
| Cough | 59 (59%) | 8 (40%) | 12 (44.5%) | 39 (67.2%) | 0.09 | 0.35 | 0.29 |
|  |  |  |  |  |  |  |  |
| Respiratory rate, /min^†^ | 32.4 (8.67) | 21.4 (2.06) | 28.3 (5.80) | 37.7 (6.27) | **<0.0001** | **<0.0001** | **<0.0001** |
| SpO2, %^†^ | 87.5 (13.9) | 98.4 (1.14) | 93.5 (4.23) | 81.4 (15.4) | **<0.0001** | **<0.0001** | **<0.0001** |
|  |  |  |  |  |  |  |  |
| Total count, 10^9^ per L^†^ | 10.2 (5.98) | 8.57 (5.47) | 9.71 (4.09) | 10.8 (6.67) | 0.38 | 0.5 | 0.47 |
| Neutrophil count, 10^9^ per L^†^ | 8.18 (5.28) | 5.57 (4.33) | 7.48 (3.64) | 9.29 (5.8) | **<0.001** | **0.003** | **<0.001** |
| Lymphocyte count, 10^9^ per L^†^ | 1.27 (0.99) | 1.88 (1.31) | 1.46 (0.7) | 1.01 (0.9) | **<0.001** | **0.01** | **<0.001** |
| **Ventilation Data** | | | | | | | |
| Non-invasive ventilation only | 39 (39%) | 0 (0%) | 0 (0%) | 39 (67.2%) | N/A | N/A | N/A |
| Invasive ventilation | 19 (19%) | 0 (0%) | 0 (0%) | 19 (32.8%) | N/A | N/A | 0.001 |
| Duration of ventilation ^‡^ | 7.5 (6-14) | 0 | 0 | 7.5 (6-14) | N/A | N/A | N/A |

Data are presented as number (percentage) and p value is obtained from Chi-square test.

* p value is obtained from Chi-square test/Fisher’s exact test (less cell count) and Yates continuity correction (zero cell) for categorical data, one-way ANOVA for continuous data and Kruskal Wallis test for skewed data.

† Data are presented as mean (SD) and p value is obtained from t test.

‡Data are presented as median (IQR), and p value is obtained from nonparametric Mann-Whitney U test.

N/A: p value is not applicable due to very less number in one category/data is not available for one or two or three groups

**Supplementary Table S2:** ROTEM parameters at day 1 and 5.

| **Parameter** | **Day 1** | | | | | | | |
| --- | --- | --- | --- | --- | --- | --- | --- | --- |
|  | **Reference Ranges** | **Mild**  **(n=20)** | **Moderate-Severe**  **(n=22)** | **Critical**  **(n=58)** | **p value*** | **p value** | | |
|  |  |  |  |  | **All three group** | **Mild vs Moderate-Severe** | **Moderate-Severe vs Critical** | **Critical vs Mild** |
| **Global hemostasis** | | | | | | | | |
| **MODIFIED EXTEM**^††^ |  |  |  |  |  |  |  |  |
| CT, s | 324–565 | 298 (63.2) | 352 (121) | 428 (293) | **0.007** | 0.09 | 0.24 | 0.053 |
| CFT, s^†^ | 112–224 | 94(80–123) | 94(71.5–132) | 102(86–167) | 0.26 | 0.46 | 0.26 | 0.144 |
| Alpha angle, degree | 50–68 | 70 (6.13) | 69.0 (9.56) | 65.7 (11.9) | 0.21 | 0.69 | 0.25 | 0.130 |
| MCF, mm | 55–66 | 65.2 (7.92) | 65.6 (9.48) | 64.5 (9.03) | 0.86 | 0.86 | 0.62 | 0.770 |
| ML, % | 0–15 | 7.05 (4.48) | 8.18 (4.22) | 5.05 (3.97) | **0.007** | 0.41 | **0.003** | 0.064 |
| **FIBTEM** |  |  |  |  |  |  |  |  |
| CT, s^†^ | 233–426 | 330(254–352) | 345(277–423) | 375(294–463) | **0.02** | 0.14 | 0.12 | **0.007** |
| MCF, mm | 4.4–18.8 | 27 (13.1) | 29.1 (13.8) | 24.9 (8.59) | 0.29 | 0.48 | 0.11 | 0.428 |
|  | **Day 5** | | | | | | | |
| **Global hemostasis** | | | | | | | | |
| **MODIFIED EXTEM**^††^ |  |  |  |  |  |  |  |  |
| CT, s | 324–565 | 319 (102) | 339 (102) | 495 (389) | **0.01** | 0.59 | 0.07 | 0.090 |
| CFT, s^†^ | 112–224 | 93(76–105) | 90.5(74.2–101.7) | 112 (87–166) | **0.02** | 0.30 | 0.06 | **0.016** |
| Alpha angle, degree | 50–68 | 72.5 (4.22) | 70.0 (9.76) | 63.5 (15.2) | **0.03** | 0.38 | 0.11 | **<0.0001** |
| MCF, mm | 55–66 | 68.1 (6.73) | 66.5 (8.7) | 63.9 (9.97) | 0.24 | 0.57 | 0.34 | 0.123 |
| ML, % | 0–15 | 7.67 (4.29) | 8.25 (3.59) | 3.28 (3.29) | **<0.0001** | 0.68 | **<0.0001** | **<0.0001** |
| **FIBTEM** |  |  |  |  |  |  |  |  |
| CT, s^†^ | 233–426 | 323(256–365) | 329(288–404) | 406(339–553) | **0.003** | 0.65 | **0.01** | **0.004** |
| MCF, mm | 4.4–18.8 | 30 (16.5) | 28.5 (11.6) | 22.6 (7.78) | **0.03** | 0.79 | **0.02** | 0.123 |

Data are presented as mean (SD) and p value is obtained from t test. * p value is obtained from one-way ANOVA for continuous data and Kruskal Wallis test for skewed data.

^†^ Data are presented as median (IQR) and p value is obtained from non-parametric Mann-Whitney U test. VWF Ag = von Willebrand factor antigen, PFA 200 = platelet function analysis, APTT = Activated partial thromboplastin time, CT=clotting time, CFT=clot formation time, MCF=maximum clot firmness, ML=maximal lysis. ††EXTEM reagent was modified by using low tissue factor concentration to reflect physiological conditions.

**Supplementary Table S3:** Cox regression model for markers associated with hospital mortality.

|  | **Cut off value^*^** | **Hazards Ratio (95% Confidence Interval)** | **p value** |
| --- | --- | --- | --- |
| **Day 1** |  |  |  |
| Soluble thrombomodulin, ng/mL | 5.16 | 1.80 (0.74–4.39) | 0.2 |
| VWF antigen, U/dL | 254 | 2.29 (0.66–7.89) | 0.19 |
| D-dimer, ng/mL | 969 | 1.47 (0.54–3.99) | 0.45 |
| Soluble P-selectin, ng/mL | 24.5 | 1.07 (0.38–3.02) | 0.9 |
| Beta-thromboglobulin, ng/mL | 0.69 | 2.23 (0.51–9.71) | 0.29 |
| **Day 5** |  |  |  |
| Soluble thrombomodulin, ng/mL | 4.6 | 6.40 (0.84–48.7) | 0.07 |
| VWF antigen, U/dL | 315 | 2.33 (0.75–7.28) | 0.14 |
| D-dimer, ng/mL | 1103 | 3.84 (0.86–17.08) | 0.08 |
| Soluble P-selectin, ng/mL | 41.7 | 1.41 (0.48–4.12) | 0.53 |
| Beta-thromboglobulin, ng/mL | 1.5 | 0.81 (0.26–2.52) | 0.71 |

VWF Ag=von Willebrand factor antigen.

^*^ Cut off value calculation: Receiving operating curve (ROC) analysis was first performed using in-hospital mortality as the classification variable and biomarker level as the prognostic variable. Area under the curve (AUC) was calculated using ROC. The optimal cut off for each marker was determined using the highest Youden Index.
